# Description of organ-specific phenotype, and functional characteristics of tissue resident lymphocytes from liver transplantation donor and research on immune tolerance mechanism of liver

**DOI:** 10.18632/oncotarget.24514

**Published:** 2018-02-15

**Authors:** Yunpeng Shi, Ping Zhang, Guangyi Wang, Xingkai Liu, Xiaodong Sun, Xin Zhang, Haijun Li, Jun Qi, Lei Ding, Ting Li, Ruoyan Zhang, Yuguo Chen, Jianpeng Zhou, Guoyue Lv, Zhengkun Tu

**Affiliations:** ^1^ Department of Hepatobiliary and Pancreatic Surgery, The First Hospital of Jilin University, Changchun, Jilin 130021, P.R. China; ^2^ Department of Surgery, Institute of Translational Medicine, The First Hospital of Jilin University, Changchun, Jilin 130061, P.R. China; ^3^ Department of Emergency Surgery, Jilin Province People's Hospital, Changchun, Jilin 130021, P.R. China

**Keywords:** liver transplantation, intrahepatic lymphocytes, liver immune tolerance

## Abstract

**Aim:**

Prior to transplantation, Donation after Cardiac Death (DCD) liver transplantation livers are perfused with preservation solution. Therefore, this provides an abundant source of human liver lymphocytes, as well as mesenteric lymph node and spleen for the study of lymphocyte subset diversity in the peripheral blood, lymph node, spleen and liver.

**Methods:**

Lymphocyte subsets were isolated and purified from peripheral blood, lymph node, spleen and liver perfusion, the phenotypic and functional analysis of the tissue resident lymphocyte were performed by flow cytometry.

**Results:**

In a direct comparison between blood, liver, lymph node and spleen cells from liver transplantation donors, the abundance of natural killer (NK) cells, CD3^+^CD56^+^NKT (NT) cells and CD8^+^ T cells in intrahapatic lymphocytes (IHL) did not match what was present in peripheral blood and other peripheral lymphoid organs. The activation state of peripheral blood-derived lymphocytes was significantly different from lymph node-, spleen- and liver-derived cells. Intriguingly, NK cells, CD4^+^ T cells, and CD8^+^ T cells from liver perfusion display more suppressive characteristics, that is, express and produce more anti-inflammatory cytokine interleukin (IL)-10, less inflammatory cytokine interferon (INF)-γ.

**Conclusion:**

Our findings imply that different tissues entail resident lymphocyte subsets with a distinct phenotype and function considering the organ is well vascularized, particularly in liver. It is better to understand the mechanism of liver immune tolerance.

## INTRODUCTION

Lymphocytes migration from the blood across the non-lymphoid and lymphoid tissues through lymphatic system secured the parenchymal cells, tissues and organs is a prerequisite for immune surveillance [1, 2]. Depending on their distinct functions, the resident lymphocytes in different organs and the migration pathways of lymphocytes in the lymphoid system and peripheral non-lymphoid tissue can vary [3–6]. The chemokine system orchestrates lympocytes migration and positioning in homeostasis, in acute inflammation and during the generation and regulation of adoptive primary and secondary immune responses [7]. Bone marrow-derived NK cell precursors migrate from the bone marrow through the blood to the spleen, liver, and many other organs by the expression of chemotactic receptors and adhesion molecules, and undergo a maturation process that leads to the acquisition of their effector functions. Thus, NK cells populate different tissues and organs, including peripheral blood (PB), lymph nodes (LN), spleen, liver, and other secondary lymphoid organs [8, 9].

NK cells are differentiated from NK cell precursors (NKPs). Bone marrow hematopoietic stem cells can differentiate into NKPs, and early lymphoid precursors (ELPs) can also differentiate into NKPs. In recent years, studies have confirmed the presence of NKPs in the liver, lymph nodes, spleen, indicating that all these tissues and organs can be the site of NK cell development and differentiation. During the development and differentiation of NK cells, bone marrow-derived NKPs can migrate to the thymus, lymph nodes, liver, spleen and other sites; thymus-derived NKPs can migrate to the lymph nodes and then further differentiate. In addition, NKPs are also migrated between lymph nodes and spleen [10, 11]. But, the distribution of NK cells is not static because these cells can recirculate between organs. The characteristics of NK cells in different organs may reflect their ability to adapt to different micro-environments. CD3^+^CD56^+^ natural T (NT) cells are a subset of human T lymphocytes expresses NK cell-associated receptor CD56 [12], which comprise approximately 5 to 15% of the peripheral T-cell pool and up to 50% of T cells within the liver environment [13]. Functionally, NT cells display properties of both NK cells and T cells, are capable of NK-like major histocompatibility complex (MHC)-unrestricted cytotoxicity and TCR/CD1d-mediated cytotoxicity and secretion of cytokines. Studies in mice have revealed an important role for tissue resident memory CD4^+^ and CD8^+^ T cells in protective immunity to site-specific pathogens [14–15], and these tissue-retained memory populations do not recirculate [16–18].

More recent studies in human T cells in lymphoid and mucosal tissues obtained from individual organ donors showed that tissue-intrinsic compartmentalization of naive, effector and memory T cell subsets conserved between diverse individuals [19]. Initial T cells are those cells that have left the thymus but have not yet received antigen stimulation. These cells function as immune monitors through the recycling of blood and secondary lymphoid organs. In the lymph nodes, antigen-bearing dendritic cells (DCs) from peripherally infected sites activate initial T cells to differentiate into effector T cells. When initial T cells differentiate into effector T cells, their functional and migration properties have changed [20, 21]. According to the expression of phenotype CD45 subtypes, human T cells can be divided into naive T cells (CD45RA+) and memory T cells (CD45RA-), and according to their homing characteristics and effector functions, CD4 + and CD8+ T cells are divided into central memory T cells (TCM) and effector memory T cells (TEM). TCM and TEM are different in their function and mobility. Regulatory T cells (Tregs) refer to T cells that recognize the TCR antigen peptide that is raised by MHC molecules and exert some immunosuppressive function. It can be divided into natural regulatory T cells (nTregs) and inducible regulatory T cells (iTregs). nTreg cells play a crucial role in maintaining normal peripheral immune tolerance and immune response homeostasis. iTreg cells play an important role in tumorigenesis and development and have a high degree of immunosuppressive function, which can down-regulate the anti-tumor immune response in the body [22, 23]. Most of studies in human lymphocytes concentrate on peripheral blood because of ethic limitation, the distribution and function of human lymphocytes and subsets in lymphoid and non-lymphoid tissues remain to be well elucidated.

In routine clinical practice, peripheral blood as an easily accessible organ system is often used to screen for pathological conditions. However, lymphocytes in the peripheral blood represent only about 2% of the total numbers of lymphocytes in humans [1]. Thus, it is important to compare lymphocyte subsets in the peripheral blood with lymphoid and non-lymphoid tissue before extrapolating the data from peripheral blood analysis to the situation in other organ systems. Since tissue samples of healthy normal people without treatment are not readily available, the difficulties in elucidating this question are obvious. Although minimally-invasive biopsies can be taken of most organs, those biopsies are often taken to verify a pathologic condition.

Liver transplantation is a successful treatment for end-stage liver disease [24]. Liver transplantation is nowadays a routine procedure for the treatment of terminal liver failure, and often represents the only chance of a cure. Immune responses in the liver are biased towards tolerance, and this concept comes from early experiment in orthotopic liver transplantation [25]. However, the mechanism of this liver transplantation tolerance remains to be well elucidated. Prior to transplantation, donor livers are perfused with preservation solution. Therefore, this provides an abundant source of normal human liver lymphocytes, as well as mesenteric lymph node and spleen for the study of lymphocyte subset diversity in the peripheral blood, lymph node, spleen and liver. The overall objective of this study was to test whether liver-derived lymphocytes were significantly different from lymph node-, spleen- and blood-derived cells. This could be helpful to explain the mechanism of human liver immune tolerance.

## RESULTS

### Clinical characteristics of the liver donors in the study

In our study, 21 deceased organ donors diagnosed for brain death and cardiac death at the First Hospital, Jilin University were used to study lymphocyte sub-populations in lymphoid and non-lymphoid tissues (approved by Ethics Committee of the First Hospital, Jilin University). Donors were between the ages of 23–62 yrs (median age was 45) and included 17 males and 4 females. The causes of death included: 19 donors had traumatic brain injuries (TBI), one donor had a cerebral tumor and one donor had moyamoya disease (Table 1). All donors were human immunodeficiency virus (HIV)/hepatitis B virus (HBV)/hepatitis C virus (HCV)-negative. Two donors had slightly elevated liver enzymes indicative of possible liver injury glutamic-pyruvic transaminase (ALT) > 40.

**Table 1:** Clinical characteristics of the liver transplantation donors in the study

Donors	Sex	Age (y)	ALT (IU/ml)	HBsAg (S/CO)	HBsAb (mIU/ml)	HCV Ab(S/CO)	Brain death cause
Donor 1	M	56	19	0.01	766	0.04	Intracranial injury
Donor 2	M	57	31	0	846	0.03	Cerebral tumor
Donor 3	M	50	12	0.32	>1000	0.05	Intracranial injury
Donor 4	M	50	15	0.013	0.306	0.125	Subarachnoid hemorrhage
Donor 5	M	56	25	0.01	0.01	0.05	Subarachnoid hemorrhage
Donor 6	M	62	14	0.02	0.1	0.04	Cerebellar hemorrhage
Donor 7	M	42	22	0.01	3.2	0.125	Basal ganglia hemorrhage
Donor 8	M	23	15	0.53	>1000	0.05	Subarachnoid hemorrhage
Donor 9	M	51	22	0.02	>1000	0.06	Subarachnoid hemorrhage
Donor 10	F	39	15	0	>1000	0.03	Subarachnoid hemorrhage
Donor 11	M	42	18	0.01	659	0.04	Subarachnoid hemorrhage
Donor 12	F	57	32	0.04	0.1	0.08	Subarachnoid hemorrhage
Donor 13	M	55	26	0.63	31	0.03	Intracranial injury
Donor 14	F	43	16	0.23	0.2	0.02	Intracranial injury
Donor 15	M	45	11	0.19	0.5	0.5	Severe craniocerebral injury
Donor 16	M	45	14	0.31	>1000	0.02	Hernia cerebri
Donor 17	M	26	50	0.14	0	0.03	Acute subdural hematoma
Donor 18	M	34	95	50.11	0	0.01	Acute subdural hematoma
Donor 19	F	41	10	0.01	3.86	26.9	Moyamoya disease
Donor 20	M	44	28	0.11	42.9	0.01	Severe craniocerebral injury
Donor 21	M	57	21	0.25	0.4	0.03	Subarachnoid hemorrhage

### Liver perfusate leukocytes are representative of IHL

**Figure 1:**
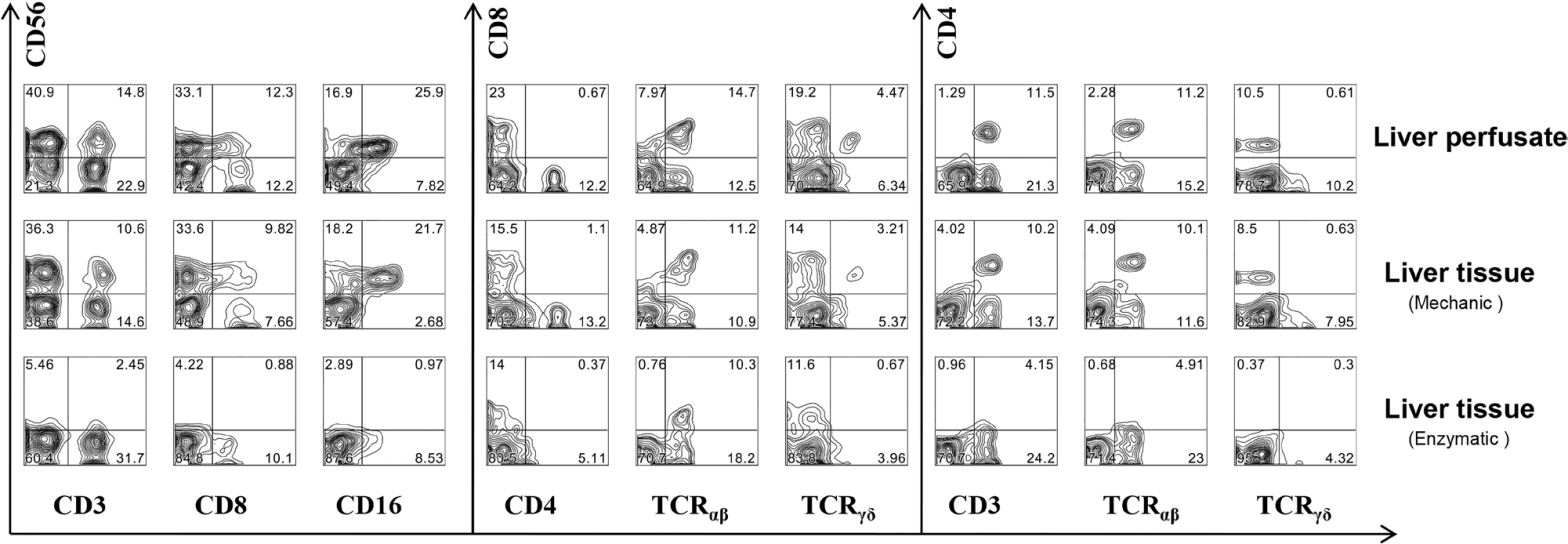
The comparison of intrahepatic lymphocytes subsets isolated from liver perfusion and liver tissue A complete DCD transplantation donor liver was released for research by the organ procurement organization after a transplant operation was aborted, and there was insufficient time to locate an alternative recipient. The availability of this liver allowed us to make a back-to-back comparison of different techniques of IHL isolation. Intrahepatic lymphocytes subsets isolated from liver perfusion and liver tissue before perfusion in the donor by traditional mechanical homogenization and enzymatic digestion methods.

A complete donor liver was released for research by the organ procurement organization after a transplant operation was aborted, and there was insufficient time to locate an alternative recipient. The availability of this liver allowed us to make a back-to-back comparison of different techniques of IHL isolation. Figure 1 shows the lymphocyte subsets eluted from this liver by three different techniques: portal vein flush with organ preservation solution, mechanical homogenization, and enzymatic digestion followed by mechanical homogenization. The portal vein flush gave the best preservation of cell phenotypes, and particularly of the CD56^+^, CD8^+^, CD4^+^, TCRαβ^+^ and TCRγδ^+^ subsets, compared to the mechanical methods. αβ + T cells include cytotoxic T cells, NKT cells and TH cells (the TCR doublet peptide of T cell receptor consists of α chain and β chain), while the corresponding γδ + T cells (the double-peptide chain of t-cell receptor consists of γ chain and δ chain) have not been given much attention in the past due to their low proportion in the peripheral blood (accounting for only 5–10% of the total number of mature T-cells). However, this group of cells is particularly enriched in the liver, reaching up to 25% of the total number of local T cells, and plays an important immune surveillance role in the liver [26]. Relative to αβ + T cells, γδ + T cells have many specific innate immune system properties: they are highly diverse, has no major histocompatibility complex (MHC) restriction and does not rely on antigen handling and presentation, suggesting that γδ + T cells act as the first line of defense in fighting infections. γδ + T cells can develop independently of the thymus. They are a group of T cells with both innate immunity and specific immunity. They have dual functions of killing and regulating, playing an irreplaceable role in the maintenance of local immune homeostasis [27]. Surprisingly, though enzymatic isolation increased lymphocyte yield compared with mechanical isolation, some lymphocytes lost cell surface molecules completely (CD56 , CD4, and TCRγδ) or partially (CD8, CD16, and TCRαβ). Moreover, the flush technique yields a much higher number of the cells of interest than mechanical homogenization, and even enzymatic digestion. Therefore, the direct harvest of human IHL by elution from liver perfusion of liver donors allowed us to obtain a high quality sample of human liver lymphocytes without tissue dissociation or enzyme incubation.

### The frequency of lymphocyte subsets derived from peripheral blood, lymph node, spleen and liver perfusate from cadaver donors

**Figure 2:**
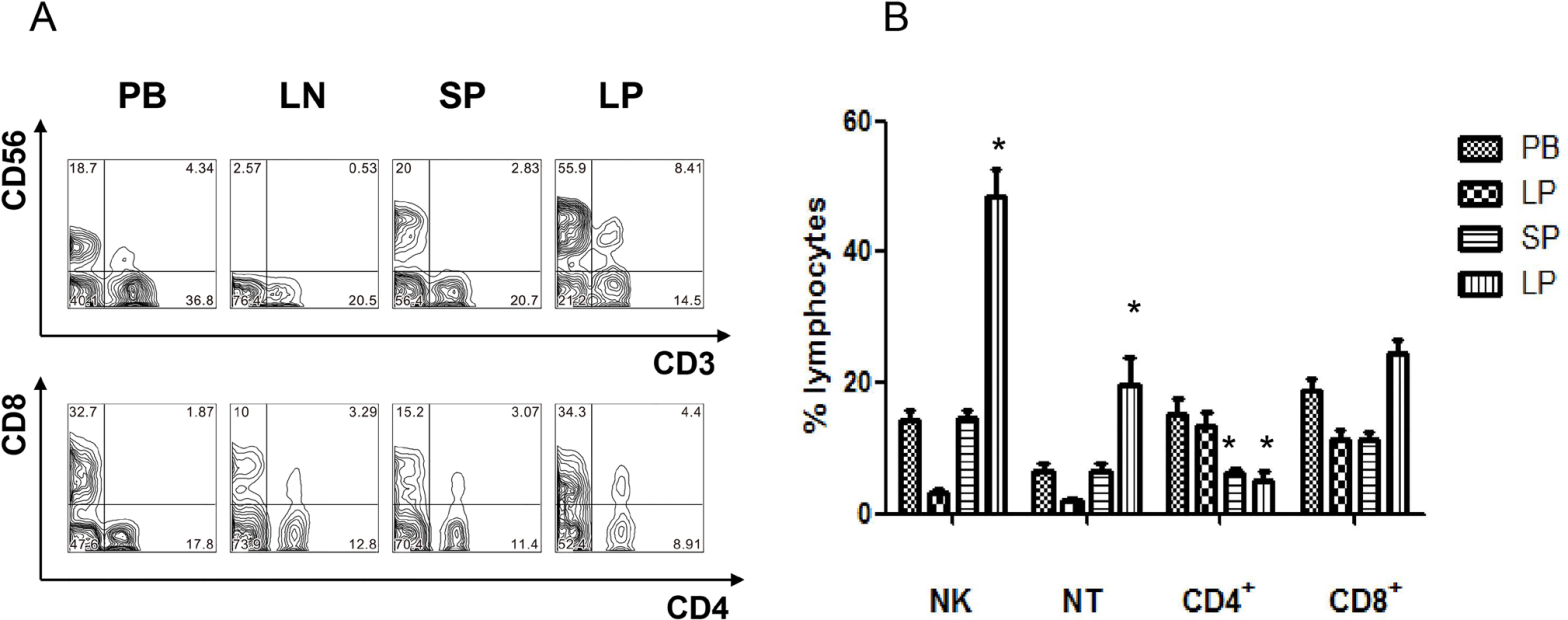
The frequency comparison of lymphocyte subsets derived from peripheral blood, lymph node, spleen, and liver perfusion of liver donors Mononuclear cells were isolated from peripheral blood, lymph node, spleen, and liver perfusion of 21 transplantation donors. The frequency of lymphocyte subsets were performed by flow cytometry. (**A**) Representative FACS data from a single donor. (**B**) Statistic analysis from 21 transplantation donors (*n* = 21, ^*^P < 0.05).

We isolated mononuclear cells from peripheral blood, lymph node, spleen, and liver perfusate and used flow cytometry to analyze the frequency of lymphocyte subsets. The results are shown in Figure 2. The (CD3^-^CD56^+^) NK cells and (CD3^+^CD56^+^) NT cells percentage of lymphocytes in liver (NK:48.62% ± 4.04, NT:19.59% ± 4.43) were significantly higher than in peripheral blood (NK:14.36% ± 1.54, *P* < 0.05; NT:6.56% ± 1.16, *P* < 0.05), lymph node (NK: 3.1% ± 0.71, *P* < 0.01; NT: 1.97% ± 0.43, *P* < 0.01) and spleen (NK: 14.58% ± 1.34, *P* < 0.05; NT: 6.4% ± 1.43, *P* < 0.05). In contrast, CD3^+^CD4^+^ T cells in liver (5.11% ± 1.35) and spleen (6.29% ± 0.64 ) were significantly lower than in peripheral blood (15.26% ± 2.37, *P* < 0.05) and lymph node (13.49% ± 2.03, *P* < 0.05). The data showed that the abundance of NK and NT cells is the characteristic of IHL, but these cells are not present in peripheral blood and other peripheral lymphoid organs.

### The phenotype of lymphocyte subsets derived from peripheral blood, lymph node, spleen and liver perfusate from liver donors

**Figure 3:**
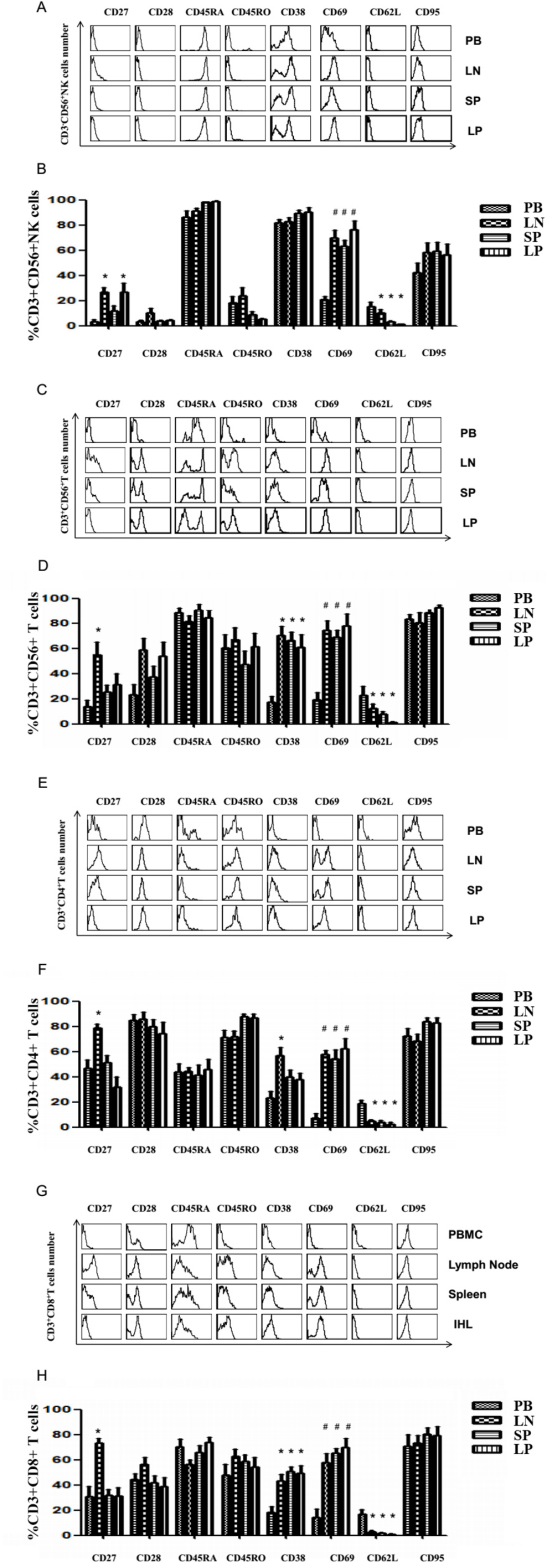
Activation markers on lymphocyte subsets among the blood cells (PBMC), lymph node cells, spleen cells and IHL from transplantation donors Mononuclear cells were isolated from peripheral blood, lymph node, spleen, and liver perfusion of 21 liver donors. The phenotypic analysis of lymphocytes subsets was also performed by gating on CD3^-^CD56^+^ NK cells, CD3^+^CD56^+^ NT cells, CD3^+^CD4^+^ T, and CD3^+^CD8^+^ T cells (**A, C, E, G**) Representative FACS data of NK cells, CD56^+^ T cells, CD4^+^, CD8^+^ T cells from a single donorin the peripheral blood, lymph node, spleen and liver perfusion. (**B, D, F, H**) Phenotypic analysis of NK cells, NT cells, CD4^+^, CD8^+^ T cells from 21 liver donors (*n* = 21, ^*^P < 0.05, ^#^*P* < 0.01).

The phenotypic analysis of lymphocyte subsets from the blood, lymph node, spleen, and liver perfusate was also performed. The results of such an analysis are reflected in the FACS histograms when gated on NK, CD56^+^ T, CD4^+^ T and CD8^+^ T cells and are shown for one representative donor (Figures 3A, 3B). Results from the group of 21 donors are summarized and statistically analyzed in Table 2 and 3. NK cells displayed an increased CD27 expression in the liver and lymph node, elevated CD69 in liver, spleen and lymph node and depressed CD62L in spleen and liver when compared to peripheral blood. Both T and NK cells express co-stimulatory and activating molecules CD27. CD27 molecules expressed on the surface of NK cells can bind to CD70 molecules on the surface of tumor cells to transduce activation signals and enhance the expression and release of perforin and granzyme B and promote the killing activity of NK cells. Meanwhile, the expression of CD27 gradually decreases with the increase of NK cell killing activity [28]. CD62L is highly expressed in the CD56hi subpopulation. Since the expression of adhesion molecules and chemokine receptors is related to the homing properties of cells, it is believed that the expression of CD62L facilitates the migration of CD56hi cells into the lymph nodes and promotes the specific immune response, therefore plays non-specific immune and specific immune role [29]. Another activated molecule, CD69, is an early sign of activation of NK cells when they are activated *in vitro*. Quiescent NK cells do not express CD69. Recent findings also show that CD69 expression in NK cells has an inhibitory effect on immune responses. By inducing the production of TGF-β, NK cell killing function and cytokine production are inhibited. However, the mouse model of removing CD69 showed strong antitumor properties, indicating that the body is self-limiting in eliminating exogenous infection and tumor cells immune response [30]. Strikingly, there were no clear-cut differences in NK cells differentiation and activation markers between liver and secondary lymphoid organs. The CD56^+^ T cells showed elevated CD27 in lymph nodes, elevated CD38 in lymph nodes, spleen and liver lymphocytes, elevated CD69 in lymph node, spleen and liver lymphocytes, and depressed CD62L in liver lymphocytes when compared to peripheral blood. Examination of CD4^+^ T lymphocytes revealed elevated CD27 and CD38 in the lymph node, elevated CD69 in lymph node, spleen and liver, and depressed CD62L in the lymph node, spleen, and liver lymphocytes when compared to peripheral blood. CD27 molecules play an important role in T cell activation, CTL differentiation and cell function, B cell differentiation and Ig synthesis and NK cytotoxicity regulation. In the lymphatic system, activated lymphocytes show high levels of CD38. In the lymphatic system, high levels of CD38 are expressed on activated lymphocytes. In peripheral blood, CD38 is expressed in NK, T, B cells; most of normal bone marrow cells express CD38. The distribution of CD38 in tissues is associated with glucose, and tissues that express more glucose also express relatively high levels of CD38. CD69 is a multidirectional immunomodulatory molecule that plays an important role in the activation and differentiation of many hematopoietic cells. CD69 is not expressed in quiescent T cells and NK cells, and is an important molecule involved in cell activation. In B cells, LPS can induce the expression of CD69. The biological functions of CD62L mainly include: 1, mediating the homing of lymphocytes to surrounding lymph nodes; 2, playing an important role in the removal and migration of lymphocytes, and then participating in the inflammatory reaction and immune response of the body. It has been reported in the literature that CD62L participates in organ transplant rejection [31].The most clear-cut difference between the liver and the peripheral lymphoid organs was an elevation in CD27 for lymph node-derived lymphocytes compared to liver lymphocytes. For CD8^+^ T cells, there was an elevated CD27 and CD38 in lymph node, elevated CD38 in the spleen and liver, elevated CD69 in all three organs, and depressed CD62L in lymph node, spleen and liver lymphocytes.

**Table 2:** NK and NK-T cells from multiple lymphoid organs

	PB (*n* = 21)	LN (*n* = 21)	SP (*n* = 21)	LP (*n* = 21)
NK				
CD16+	72.77 ± 6.54	18.05 ± 2.35^*^	33.76 ± 3.54^*^	28.14 ± 1.28^*^
CD27+	3.12 ± 1.55	26.78 ± 3.77^*^	11.86 ± 3.87	26.99 ± 6.81^*^
CD28+	3.17 ± 1.04	10.50 ± 3.53	3.83 ± 0.68	4.50 ± 0.54
CD45RA+	86.36 ± 4.86	91.20 ± 1.96	98.15 ± 0.33^*^	98.86 ± 0.38^*^
CD45RO+	18.20 ± 5.28	23.61 ± 6.62	8.96 ± 2.29	5.39 ± 0.59
CD38+	81.90 ± 2.57	82.68 ± 3.14	89.44 ± 2.53	90.11 ± 3.75
CD69+	20.91 ± 2.41	69.66 ± 5.98^*^	63.39 ± 4.39^*^	76.38 ± 6.83^*^
CD62L+	15.12 ± 3.67	10.33 ± 2.74	3.52 ± 0.49^*^	1.17 ± 0.31^*^
CD95+	42.25 ± 7.75	58.16 ± 7.81	59.58 ± 6.68	56.35 ± 8.46
NKT				
CD27+	13.99 ± 4.90	55 ± 9.72^*^	25.53 ± 5.36	31.11 ± 8.57
CD28+	23.35 ± 8.0	58.78 ± 8.85	37.47 ± 8.50	53.66 ± 11.01
CD45RA+	88.49 ± 3.72	81.45 ± 4.32	90.83 ± 4.35	84.55 ± 5.95
CD45RO+	60.26 ± 10.74	67 ± 9.28	47.51 ± 10.55	61.24 ± 10.40
CD38+	17.34 ± 4.29	70.49 ± 6.60^*^	66.2 ± 6.43^*^	61.04 ± 9.61^*^
CD69+	19.17 ± 5.61	74.23 ± 7.81^*^	68.79 ± 5.33^*^	77.81 ± 9.44^*^
CD62L+	22.97 ± 6.78	12.22 ± 3.80	7.72 ± 2.04	1.50 ± 0.38^*^
CD95+	83.44 ± 3.16	80.53 ± 7.77	88.53 ± 2.46	92.83 ± 1.86

**Table 3:** Classical T cells from multiple lymphoid organs

	PB (*n* = 21)	LN (*n* = 21)	SP (*n* = 21)	LP (*n* = 21)
CD4+				
CD27+	46.93 ± 6.53	78.89 ± 3.16^*^	51.15 ± 5.71	31.80 ± 7.90†
CD28+	85.05 ± 4.44	86.03 ± 5.33	79.63 ± 5.49	74.55 ± 8.92
CD45RA+	44.08 ± 6.17	44.15 ± 3.36	41.18 ± 8.25	45.74 ± 7.89
CD45RO+	71.56 ± 5.46	71.99 ± 4.43	87.78 ± 2.04	86.63 ± 3.23
CD38+	23.51 ± 4.88	56.75 ± 6.65^*^	40.06 ± 5.33	37.83 ± 4.82
CD69+	7.28 ± 3.58	57.95 ± 3.01^*^	54.29 ± 6.85^*^	62.41 ± 7.84^*^
CD62L+	18.63 ± 2.91	4.68 ± 1.33^*^	4.02 ± 1.32^*^	2.39 ± 1.38^*^
CD95+	72.16 ± 6.08	68.41 ± 5.32	83.83 ± 3.15	82.86 ± 4.08
CD8+				
CD27+	30.71 ± 8.3	73.3 ± 3.46^*^	31.84 ± 4.56†	31.4 ± 6.55†
CD28+	44.21 ± 4.67	56.1 ± 5.83	41.61 ± 5.78	38.8 ± 7.2
CD45RA+	70.46 ± 5.95	56.48 ± 3.42	66.01 ± 5.17	73.86 ± 4.07
CD45RO+	47.78 ± 8.54	62.71 ± 5.83	58.91 ± 5.12	54.3 ± 7.65
CD38+	18.43 ± 4.28	43.35 ± 5.01^*^	50.9 ± 3.21^*^	49.33 ± 5.84^*^
CD69+	14.33 ± 6.54	57.64 ± 7.17^*^	65.51 ± 3.11^*^	69.89 ± 6.79^*^
CD62L+	16.94 ± 3.54	2.83 ± 1.01^*^	1.78 ± 0.61^*^	0.99 ± 0.39^*^
CD95+	70.63 ± 9.05	73.43 ± 5.76	80.18 ± 5.35	79.56 ± 6.54

In summary, our results indicate the differences in lymphocyte activation markers between the lymph nodes, spleen and liver were minor; in contrast, all of these tissues contained lymphocytes that expressed more activation markers than the peripheral blood. This was true for NK cells, NK-T cells, CD4^+^ T cells and CD8^+^T cells.

### Detection of IL-10 and IFN-γ producing liver resident lymphocytes

Studies have revealed that most of the immunocytes are capable of inducing both tolerance and immunity. Hepatic lymphocytes, including NK cells, NK-T cells, and T cells may play a key role in liver tolerance. Given that the activation state of liver resident lymphocytes is distinct from peripheral blood, but minor from lymph node and spleen, we thus investigated whether liver resident lymphocytes display more suppressive characteristics than that from peripheral blood. NK cells, CD4^+^ T cells, and CD8^+^ T cells were purified from peripheral blood and liver perfusion.

**Figure 4:**
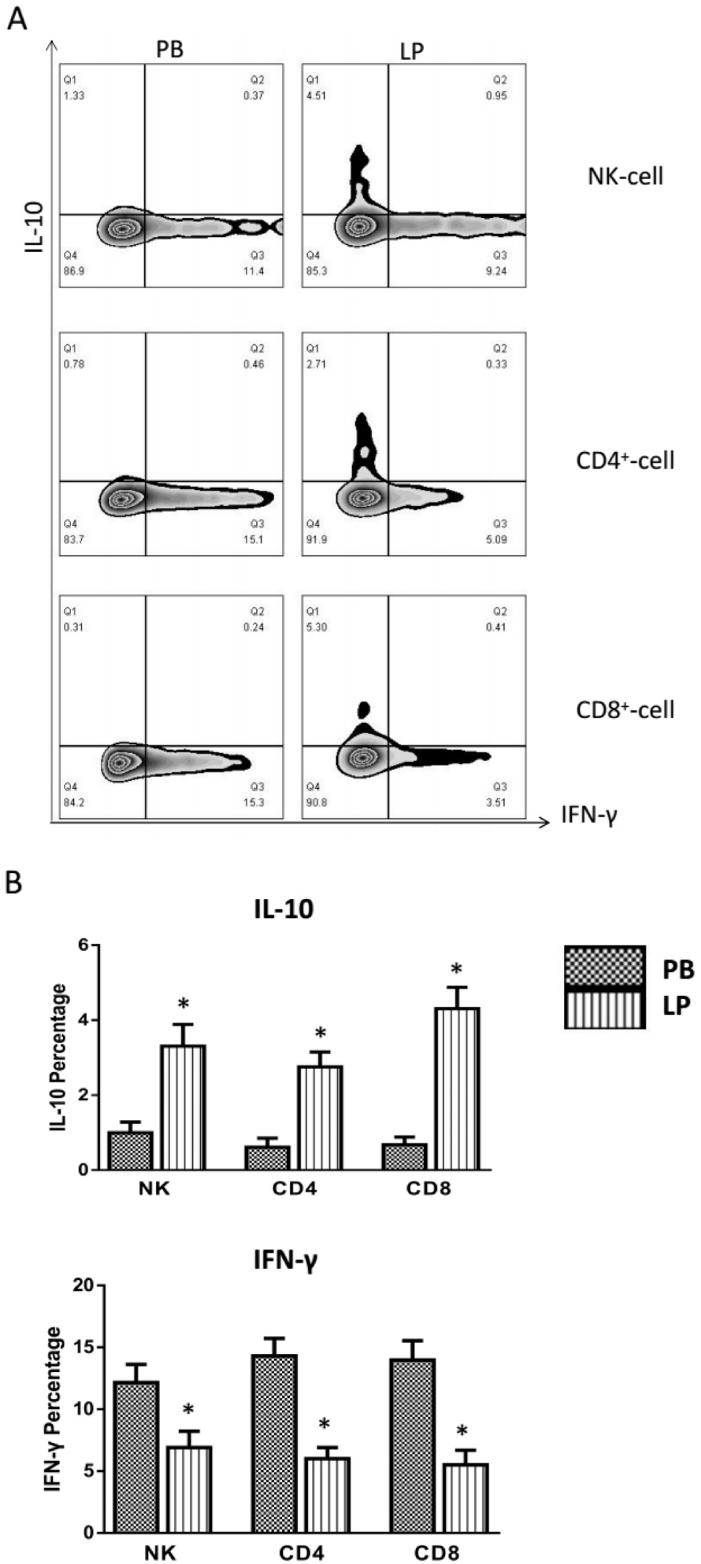
Functional analysis of the cytokines of INF-γ and IL-10 secretion from the lymphocyte of the peripheral blood and donor liver perfusion Our phenotypic analysis was performed by gating on CD3–CD56+ NK cells, CD3+CD4+ T, and CD3+CD8+ T cells. Representative IFN-γ and IL-10 expression data of NK cells, CD4+, CD8+ T cells from a single donor in the peripheral blood and liver perfusion were analyzed in **A**. Functional analysis of inflammatory and anti-inflammatory cytokine expression of NK cells, CD4+, CD8+ T cells from 21 liver donors (*n* = 21, ^*^P < 0.05, ^#^*P* < 0.01) was performed in **B**.

To further investigate if liver resident lymphocytes with distinctive phenotype and distribution lead to liver tolerance, we detected that the IL-10 produced liver resident lymphocytes, and compared with that of peripheral blood. NK, CD4^+^ and CD8^+^ T cells were isolated and purified from peripheral blood and liver perfusate. NK cells were stimulated with phytohemagglutinin (PHA), and CD4^+^ T cells and CD8^+^ T cells were stimulated with CD3 and CD28 antibodies. IFN-γ and IL-10 expression and production were determined by intracellular cytokines staining. The results show that the expression and production of IL-10 on liver resident NK cells, CD4^+^ T cells and CD8^+^ T cells are significantly higher than that from peripheral blood. In contrast, the expression and production of IFN-γ are significantly lower than that from peripheral blood (Figure 4A).

The results show that the IL-10 percentage produced of lymphocytes in (PB) peripheral blood (NK cells: 1.00% ± 0.06 *P* < 0.05, CD4^+^ T cells: 0.61% ± 0.05 *P* < 0.05, CD8^+^ T cells: 0.68% ± 0.04 *P* < 0.05) were significantly lower than in (LP) liver perfusate (NK cells: 3.31% ± 0.13 *P* < 0.05, CD4^+^ T cells: 2.76% ± 0.09 *P* < 0.05, CD8^+^ T cells: 4.31% ± 0.12 *P* < 0.05). In contrast, the IFN-γ expression and production percentage in (PB) peripheral blood (NK cells: 12.16% ± 0.32 *P* < 0.05, CD4^+^ T cells: 14.31% ± 0.31 *P* < 0.05, CD8^+^ T cells: 13.97% ± 0.34 *P* < 0.05) were significantly higher than in (LP) liver perfusate (NK cells: 6.91% ± 0.29 *P* < 0.05, CD4^+^ T cells: 6.01% ± 0.20 *P* < 0.05, CD8^+^ T cells: 5.50% ± 0.26 *P* < 0.05) (Figure 4B). The data showed that liver resident lymphocytes express and produce more anti-inflammatory cytokine IL-10, less inflammatory cytokine INF-γ than peripheral blood.

## DISCUSSION

Organ donors dying of traumatic brain injuries (TBI) yield organs that function better than donors who die from other causes [32]. We here used tissue from TBI cadavers to characterize lymphocyte sub-populations in lymphoid and non-lymphoid tissues. Among the 21 donors involved in our study, brain death caused by traumatic brain injuries occurred in 19 donors; one donor had a cerebral tumor and one donor had moyamoya disease and the average age was 45 yrs (range from 23 to 62 yrs). Although younger donors are more likely to die from trauma, multivariate analyses have shown that both donor age and traumatic cause of death are independently associated with better organ function after transplant [33].

Our results first showed that LP leukocytes are representative of IHL, and has obvious advantages over mechanical homogenization of tissue either with or without enzymatic digestion (Figure 1). There is a recognized loss of CD56^+^, CD8^+^, CD4^+^, TCRαβ^+^ and TCRγδ^+^ cell subsets presumably due to cleavage of this cell surface antigen by enzymatic digestion. Our experiments confirm the notion that these cell subsets are more easily lost in whole tissue extracts. It is evident from this study that simple flushing yields far greater numbers of these cell subsets than homogenization with or without enzymatic digestion.

In steady-state conditions, the number and distribution of lymphocyte populations are under homeostatic control. The primary goal of the cells of the immune system is not only to ensure their own growth and survival, but to respond against external threats in local tissues. So it is important to isolate and characterize lymphocytes in different tissues under normal and abnormal conditions [34]. Our previous investigations of lymphocyte have been limited to cells derived from the human liver and peripheral blood in living donor liver transplants [35]. We broadened this comparison of IHL to include not only peripheral blood, but also mesenteric lymph nodes and the spleen by collecting tissues from DCD donors. In this four-way comparison, the abundance of NK and NT cells was characteristic of liver tissue but did not match what was present in other peripheral lymphoid organs. In contrast, CD4^+^ T cells in liver and spleen were significantly lower than in peripheral blood and lymph node (Figure 2). Consisting with it, the resident liver lymphocytes from mouse show both abundance of NK, CD56^+^ T and CD8^+^ subsets and an increase in the frequency of activated and apoptotic T cells [36]. This may be relevant to the role of the liver in relation to innate immunity and exposure to gut derived antigen via the portal vein.

Liver lymphocytes have been extracted from liver donor organs and analyzed for phenotypic differences from peripheral blood previously [37–39]. Now we have extended the examination of the subsets of IHL to matched secondary lymphoid organs (the spleen and lymph nodes) from human liver donors. Our results showed that NK and NT cells displayed elevated activation markers (CD69, CD27, CD38) in the liver, lymph node and spleen, compared to peripheral blood (Figure 3 and Table 2). Memory T cells are distinct from naive T cells due to their expression of CD45RAlow, CD45ROhi. Memory T cells are divided into central memory lymphocytes (TCM) (CD62Lhi) and effector memory T lymphocytes (TEM) (CD62Llow) [40, 41]. CD4^+^ and CD8^+^ T cells isolated from liver, lymph node and spleen in this study were much more CD4^+^ CD45ROhi CD62L-T cells and CD8^+^ CD45ROhi CD62L-T cells, belonging to TEM. Activated T cells, like NK and NK-T cells, are sequestered in the liver, lymph node and spleen out of the circulation (Figure 3 and Table 3). Our data suggest the liver is not so unique as previously thought in terms of the activation status of its resident lymphocytes. Previous studies examined phenotypic differences in the human liver using peripheral blood as a comparison. Our studies confirm the differences of the liver to peripheral blood, but the activation state within the liver was remarkably similar to two secondary lymphoid organs, the spleen and lymph node. Peripheral blood is uniquely enriched for resting, yet-to-be-activated lymphocytes. Naïve lymphocytes are generally considered part of the recirculating pool and are thought to be largely confined to secondary lymphoid organs, trafficking through lymph and blood [42, 43]. After entering lymph nodes, these cells take an estimated 10–12 hr to exit through the efferent lymphatics and eventually enter the blood [44]. Experiments in normal mice suggest the recruitment of lymphocyte subsets mirrors the resident population [45]. Our studies in the human differ from those in the mice in that CD62L (L-selectin) is low in lymphocyte subsets of both liver and spleen.

The liver remains at the centre of immune tolerance and immune responses. Different food products, inflammatory substances, allergens and drug metabolites constantly enter the liver through the gut or the blood stream. Under physiological conditions, the liver induces a state of immunological tolerance to these substances to prevent extreme and detrimental immune reactions.

The unique immunologic function of the liver has been attributed to the phenotype and activation status of IHL when compared to lymphocytes in peripheral blood. However, how compromised innate immunity can be regulated in the presence of cytotoxic or immunogenic IHL remains unknown. The liver is a recognized organ with immune tolerance. When most of the toxicity and foreign matter enter liver, they do not induce an immune response [36]. In addition, the liver has a certain ability to resist transplant rejection, even when the HLA mismatch the context of the transplantation [46]. However, the properties of these cells have not been properly revealed until now. The issue becomes more important because the frequency of NK cells is usually higher in the liver compared with those in other parenchymal organs [47]. In consequence, it had been reported that immunosuppressive NK cells that produced IL-10 (an anti-inflammatory cytokine) existed in murine decidua and human peripheral blood [48, 49]. It remains unclear whether immunosuppressive or immunoregulatory NK cells are present in the liver. However, the properties of these cells have not been properly revealed until now.We were inspired by the report that liver allograft was acutely rejected in IFN-γ knockout recipients [50], which is beyond our expectation, because IFN-γ plays a role of inflammatory cytokine produced by T effector cells, and it participates in graft rejection [51]. In this study, we obtained evidence that the NK cells, CD4^+^ and CD8^+^ T cells derived from livers expressed higher levels of IL-10 relative to the other organs, and the expression of IFN-γ levels is relatively lower. This is also why in comparison with other transplantation, liver transplantation has a relatively low rejection. In other words, a liver transplant itself is prone to immune tolerance. Most of the liver nonparenchymal cells (NPCs, nearly 30%), such as dendritic cells (DCs), liver sinusoidal endothelial cells, Kupffer cells, hepatic stellate cells, and regulatory T cells are responsible for the tolerogenic properties of the liver [52, 53]. One of the important factors that endow tolerogenic property of these cells is their capacity to produce significant amounts of IL-10. Liver resident lymphocytes may play an immune regulatory role in human liver, which contributes to liver immune tolerance.

TBI associated with brain death has undoubted consequences in terms of cellular trauma and immune activation *in vivo* and elevations in the levels of various cytokines in the circulation might impact on the distribution and level of activation of lymphocyte subsets in lymphoid and non-lymphoid tissues [54, 55]. One might be tempted to speculate the activation of the lymphocytes from the liver when comparing with peripheral blood, which is due to the environmental stress associated with brain death. This is not supported by the data as comparing the values associated with peripheral blood and liver perfusion in living donors to liver donors [35]. The relatively unstressed living donors behave similarly both with regard to peripheral blood lymphocytes and liver perfusion lymphocytes. The concept that immune responses in the liver are biased towards tolerance comes from early experiment in orthotopic liver transplantation.

In summary, we compared the frequency, phenotype and function of lymphocyte subsets obtained by perfusion of donor livers to peripheral blood lymphocytes and to lymphocytes derived from the lymph nodes and spleen using matched samples from liver donor organs. In a direct comparison between blood, liver, lymph node and spleen cells from liver donors, the abundance of NK and NT cells in IHL did not match what was present in other peripheral lymphoid organs. The activation state of lymphocytes was very similar in the lymph node-, spleen- and liver-derived cells. In contrast, the activation state of blood-derived lymphocytes was significantly different. The present study suggests that “normal” human liver contains an over-abundance of NK, NT and CD8^+^ T cells, but that these cells do not have a distinctive “liver-specific” pattern of activation markers and function from secondary lymphoid organs. Lymphocyte subsets from liver as well as spleen and lymph node are activated. This implies that the tissue-resident lymphocytes may be antigen-experienced/ have a memory phenotype.

This study addresses an important and timely topic in liver transplantation. It could be helpful to explain the mechanism of human liver immune tolerance. The methodology and the findings lays an important basis for future studies.We will further compare liver donor with liver explants (and corresponding blood samples) of patients with liver diseases (i.e. the recipients of the donor livers), and explore whether or not intrahepatic lymphocyte numbers and activation markers be sufficiently compared between “healthy” controls (liver donors) and patients.

## PATIENTS AND METHODS

### Ethics and consent

The China Liver Transplant Registry (CLTR) contains 86 liver transplantation centers across China, including the liver transplant center in Jilin University. All the donor organs in this report diagnosed for brain death and cardiac death at First Hospital, Jilin University were enrolled in CLTR. There is no organ transplanted from executed prisoner in the present study. All samples were obtained from deceased (and not living) individuals, the study does not qualify as “human subjects” research, as confirmed by the Institutional Review Board at Jilin University. Consent for use of donor tissues for research was obtained by transplant coordinators at Jilin University.

### Collection of samples

Twenty one organ donors diagnosed for brain death and cardiac death at the First Hospital, Jilin University were enrolled in this study (Table 1). Venous blood were withdrawn before organ procurement and tissue samples of mesenteric lymph nodes, spleen and liver were maintained in cold saline and brought to the laboratory within 2 hours following organ procurement. Liver perfusates were collected as described [35, 56–58]. Briefly, the donor aortaabdominalis and superior mesenteric vein were initially flushed with University of Wisconsin (UW) solution (CHD072117, Whitney Hui Li Medical company) at the time of exsanguination. The liver was flushed again with UW solution after excision of the organ until all blood was removed and the perfusate appeared clear, at which time the liver was placed in a container with UW solution and packed on ice for transportation.

### Cell isolation

Peripheral blood mononuclear cells (PBMCs) were isolated by Ficoll-Hypaque (17-1440-03, GE Healthcare Life Sciences) density-gradient centrifugation and resuspended in RPMI 1640 medium (R10-040-CV , CORNING) supplemented with 2 mmol/L L-glutamine, 100 IU/mL penicillin (SV30010, HyClone), 100 g/mL Streptomycin (SV30010, HyClone), 25 mmol/L HEPES(04676, Waryong) and 10% FBS(10100147, Gibco). Spleen, lymph node, and liver tissue was put on ice, washed twice in Hank’s balanced salt solution (HBSS) (14175103, Thermo Fisher) and scraped extensively with a sterile blade into homogenized suspension with or without a mixture containing 0.5 mg/ml collagenase (C1889 - 50 mg, Simga) (type IV, 312 U/mg) (Sigma-Aldrich), 0.02 μg/ml DNase I(D5025-150KU, Simga), 2% FBS, 0.6% bovine serum albumin (BSA) (A8020, Solarbio) and HBSS at 37° C. The suspension was passed through a 30 μm-nylon mesh filter to remove cell clumps and un-disassociated tissue. The filtered suspension was subjected to Ficoll-Hypaque density-gradient centrifugation. Liver perfusates were centrifuged and resuspended in RPMI 1640 complete medium, filtered through 40 μm mesh and centrifuged. The cell pellets were subjected to Ficoll-Hypaque density-gradient centrifugation.

### Flow-cytometric anlaysis

Peripheral blood lymphocytes, lymph node cells, spleen cells, and intrahepatic lymphocytes were analysed using flow cytometry as described [32]. The following antibodies were used in this study: FITC, R-phycoerythrin, Allophycocyanin-conjugated anti-CD3(555916, BD); FITC, R-phycoerythrin, PE-Cy5-conjugated anti-CD56(340410, BD); FITC, R-phycoerythrin -conjugated anti-CD4(555346, BD) and anti-CD8(557085, BD): FITC-conjugated anti-CD27 (555440, BD), CD28(16-0289-81, ebioscience), CD45RA(555488, BD), CD45RO(561887, BD), CD38(555459, BD), CD69(555530, BD), CD95(555673, BD), CD62L(555543, BD), CD16(555406, BD), CD25(560990, BD), and IgG isotype control (BD PharMingen, San Diego, CA). All data were acquired using a LSRII instrument (BD Biosciences) and were analyzed by using FlowJo (Treestar software). All samples were incubated with an Fcγ blocking antibody (14-9161-73, ebioscience) before staining.

### Cell culture

Adding anti-human CD3(16-0037-85, e-bioscience) (clone:OKT3), at a final concentration of 10 ug/ml to 100 ul PBS(0014217, BI) solution, and putting on a packet plate (352360, BD Falcon) and incubating for 3 hours at 37° C to spare; (2) Washing the plate twice with 200 ul cold PBS solution; putting mononuclear cells from peripheral blood, lymph node, spleen, and liver perfusion on the plates (each plate contains 1 million cells). (3) Adding RPMI 1640 medium, anti-human CD28 (clone:CD28) (16-0289-81, ebioscience) at a final concentration of 5 ug/ml and PHA (Phytohaemagglutinin) (L8754-50, sigma) at a final concentration of 5 ug/ml respectively, incubating for 12 hrs at 37° C in 5% CO2. With BD Golgistop protein transport inhibitor (554715, BD) containing monensin at a final concentration of 6 μg/ml, incubating for an additional 5 hrs at 37° C in 5% CO2. (4) After a total of 17 hours cell culture preparation, mononuclear cells were washed and stained with cell surface NK/CD56+ T cell markers, CD56-Pacific Blue and CD3-PECy(7557851, BD), T cells markers, CD8-Alexa FLOUR 488(557696, BD), and CD4-PECy5 (555348, BD) for 30 min. Samples were then fixed and permeabilized according to the manufacturer’s instructions and stained for intracellular IFN-γ-APC (554702, BD) and IL-10-PE (16-7108-85, ebioscience) for an additional 30 min. (5) After washing, cells were re-suspended in 1% paraformaldehyde (AR1068, Boster Wuhan) until five-color flow cytometric analysis was performed on a LSRII instrument (BD Biosciences).

### Statistical analysis

Statistical significance was determined by using the D’Agostino and Pearson omnibus normality test. Mean values were compared using either a paired *t* test (2 groups) or ANOVA (>2 groups), followed by Bonferroni correction for multiple comparison test. *P* values < 0.05 were considered significant. All statistical tests were performed using Prism software (GraphPad, San Diego, CA).

## Abbreviations

IHL: Intrahepatic Lymphocytes
PB: peripheral blood
LN: lymph nodes
NT: CD56+ natural T
iNKT: invariant NKT
PBMCs: Peripheral blood mononuclear cells
BSA: bovine serum albumin
HBSS: Hank’s balanced salt solution
DMSO: dimethyl sulphoxide
PHA: phytohemagglutinin
TBI: traumatic brain injuries
DCD: donation after cardiac death
